# Early Stress Causes Sex-Specific, Life-Long Changes in Behaviour, Levels of Gonadal Hormones, and Gene Expression in Chickens

**DOI:** 10.1371/journal.pone.0125808

**Published:** 2015-05-15

**Authors:** Magnus Elfwing, Daniel Nätt, Vivian C. Goerlich-Jansson, Mia Persson, Jonas Hjelm, Per Jensen

**Affiliations:** IFM Biology, AVIAN Behaviour Genomics and Physiology Group, Linköping University, 58183, Linköping, Sweden; Università della Tuscia, ITALY

## Abstract

Early stress can have long-lasting phenotypic effects. Previous research shows that male and female chickens differ in many behavioural aspects, and respond differently to chronic stress. The present experiment aimed to broadly characterize long-term sex differences in responses to brief events of stress experienced during the first weeks of life. Chicks from a commercial egg-laying hybrid were exposed to stress by inducing periods of social isolation during their first three weeks of life, followed by a broad behavioural, physiological and genomic characterization throughout life. Early stressed males, but not females, where more anxious in an open field-test, stayed shorter in tonic immobility and tended to have delayed sexual maturity, as shown by a tendency for lower levels of testosterone compared to controls. While early stressed females did not differ from non-stressed in fear and sexual maturation, they were more socially dominant than controls. The differential gene expression profile in hypothalamus was significantly correlated from 28 to 213 days of age in males, but not in females. In conclusion, early stress had a more pronounced long-term effect on male than on female chickens, as evidenced by behavioral, endocrine and genomic responses. This may either be attributed to inherent sex differences due to evolutionary causes, or possibly to different stress related selection pressures on the two sexes during commercial chicken breeding.

## Introduction

Stress causes cascades of both immediate and long-term changes in physiology, behaviour and gene regulation [1–3]. However, both short- and long-term responses may vary between individuals as well as between classes of individuals within a single species (for example, between sexes, or between animals with different coping styles) [1], and characterisation of such differences may greatly increase our understanding of stress effects in general. We have previously found that chronic stress in chickens (*Gallus gallus domesticus*) can cause, for example, changes in learning ability, social dominance, feeding behaviour and gene expression [4,5], and the extent of these effects were found to differ between the sexes [6]. Whether similar sex differences are also found in response to acute stress experiences (i.e, stressors acting over short periods) is less well understood.

Long-lasting biological effects of acute early stress are in general well documented. For example, in Zebra Finches, early nutritional shortages can cause life-long alterations in behaviour and reproduction, and also affect the reproductive performance in the next generation [7]. Even stress experienced as early as prenatally is known to cause long-lasting and sometimes trans-generational effects (reviewed by [8,9]). However, an important aspect of most studies on early stress is that they have mainly focussed on altricial species, where early stress can be easily imposed by, for example, maternal deprivation. Much less is known about similar effects in precocial animals, such as chickens, which are able to feed and move around on their own shortly after birth or hatch [2].

Sex differences in behaviour and physiology are widely recognized in several species, including humans [10] and chickens [11]. This is closely related to the many different strategies among animals with respect to reproduction and social behavior. Solitary life-styles are associated with different selection pressures for sex differences than gregariousness, as is the case for matriarchate versus patriarchate, and polyandry versus polygyny or polygamy, and in each of the variants, the roles of the two sexes may differ dramatically [6,12]. The chicken offers an interesting model for studies of behavioural sex differences, being highly sexually dimorphic, with an excessively ornamented male of about double the size of the more cryptic female [13]. Both sexes are sexually promiscuous, but sexual coercion by the male is often necessary for copulation to occur [14]. Given these biological differences, we hypothesised that also long-term effects of acute early stress in chickens may be sex-specific.

Long-term effects of stress may be caused at least partly by modification of brain gene expression patterns, some of which are attributable to epigenetic factors, as has been summarised in recent reviews [15,16]. For example, rats raised by mothers with lower levels of maternal care have a decreased resilience to later stressful events, mediated by modified hippocampal expression of stress related genes [17, 18]. The corticosterone response to restraint stress was also increased in adult offspring of mothers with low maternal care [19]. In some cases, stress effects on expression of specific genes have been demonstrated, for example, the estrogen receptor ERα i [20], and *BDNF*, a gene involved in many different neural processes [21].

These findings illustrate interesting phenomena from a fundamental biological perspective, as they represent important mechanisms underlying phenotypic variation within a population [22], but they may also have profound practical consequences. For example, commercially bred chickens are exposed to an extensive set of stressors during their first weeks of life, such as hatching without maternal contact, transportation, heat and cold stress, and separation from social mates [23]. Hence, understanding the details of long-term consequences of such early stress on behaviour, physiology, and gene regulation in the two sexes is important both from a perspective of animal welfare and for understanding the evolution of phenotypic variation.

The present experiment was undertaken to characterise possible sex differences in long-term reactions of chickens after exposure to a short period of stress early in their lives. In a previous paper, we reported trans-generational effects based on data from the same experiment [2]. The results we present here are thus additional, previously unpublished data, from the same animals. In the present study, we aimed to characterise the lifetime effects broadly in the exposed animals (the parental generation in the experiment reported previously), by assessing behavioural changes as well as sex hormone levels at sexual maturation. Furthermore, we used microarray methodology to study sex specific, life-long changes in stress induced hypothalamic gene expression profiles. Our hypothesis was that an early stress exposure, experienced over a limited time span, would cause different long-term effects in the two sexes, reflecting their different selection pressures in nature as well as during domestication, and paralleling those previously seen as consequence of chronic stress.

## Material and Methods

### 2.1 Ethical note

The project was approved by the Linköping Council for Ethical Licensing of Animal Experiments; ethical licence no 122–10.

### 2.2 Animals and experimental procedure

Fertilized eggs from the commercial egg-laying hybrid Hy-Line were obtained from the breeder (Swedfarm, Linghem, Sweden), and were incubated and hatched at the hatching facility of Linköping University. The eggs were all collected during one day, and since chickens lay maximum one egg per day, they all probably originated from different mothers. Hence, there were probably no siblings in the experiment.

Chickens hatch during a period of up to 30 hours, and are usually left in the incubator until all birds of a batch hatch. We therefore started our experiment two days after median hatching, so all birds would be dry and have had the possibility to feed. Chicks were assigned to a control (C) or early stress (ES) treatment, balanced for sex. The birds (94 ES and 92 C) were kept in three mixed-sex pens per group, approximately equal numbers (31–33) from each treatment in each pen, in one room under the same conditions (Light: Dark rhythm 12L:12D, ambient temperature 28°C) with free access to feed and water. Of the total 186 birds, 80 (balanced for treatment and sex) were designated for later brain gene expression analysis and were therefore kept unexposed to any behavior testing throughout life. This was done to ensure that any effects on adult gene expression would be attributable only to the differences in early treatment.

We exposed the birds to stress during the early life phase, starting as early as possible after they had habituated to their home pens, and continuing during the early chick phase (until feathering was complete). Hence, between days 4–26 the birds in the ES group were exposed to the stress treatment once per day during three consecutive weeks at randomly assigned times. The stress treatment was the same as reported in detail previously, mimicking similar stress protocols used in quail, and known to cause an acute distress reaction in the chicks (increased frequency and intensity of distress calls) [2]. Briefly, birds were removed from their home pens to an adjacent room, where they were placed individually in an empty metal mesh box allowing vocal contact, but limited sight and no physical contact with other chicks. Time in the isolation box was one hour during the first week, two hours during the second, and three hours during the third. The treatment thus contained a combination of several different stressors: handling, social isolation, thermal stress (since the temperature was 10°C lower in the cages than in the home pens), and feed and water deprivation. The reason for expanding the time for each week was to avoid that the birds would habituate to the treatment and to ascertain that there was a certain food- and water-deprivation induced also when the chicks grew larger, and therefore would be less affected by brief deprivation periods. The C group was kept unexposed to physical stressors during the corresponding time.

At day 55, all birds were moved to an adult chicken facility. The sexes were housed in separate pens, but with treatment groups mixed, under a 12L:12D light cycle at 22°C in pens measuring 3x3x3 m with perches, nests and free access to water and feed.

Behaviour tests and physiological sampling was performed on subsets of the animals, where we attempted to maintain the balance between treatment and sex in each of the tests. However, due to constraints on the test design, and a certain mortality during the growth period, the exact number of birds included in each test varied somewhat.

### 2.3 Behaviour tests

#### 2.3.1. Open Field test

At the age of four weeks, a total of 73 birds were tested in an Open Field (OF)-arena in order to assess fear and exploration, using methods detailed earlier [24,25]. Since this test was conducted shortly after the end of the stress period, it serves as a measure of the immediate responses to the treatment. The tests were conducted in four parallel arenas, and all birds could therefore be tested during a period of four days. Briefly, the chicks were placed alone in the corner (the start zone) of a dark arena, measuring 80 x 120 x 40 cm, and during 5 min following the onset of light, their behavior was recorded. Movements were recorded automatically with video, using the software EthoVision (Noldus, v 3.1). The variables obtained from the test were latency to leave the start zone, and total distance moved during the test.

#### 2.3.2 Tonic immobility

At the age of eight weeks, a tonic immobility (TI) test was performed on 77 birds. This is a widely used test for stress and anxiety in chickens [26], and the details of the method have been described earlier [24]. Briefly, the chick to be tested was carefully lifted from its home pen to a dimly lit, acoustically isolated, adjacent test room and placed on its back in a wooden cradle. The observer held the bird softly but firm with one hand over the chest for 10 s, and if the bird remained motionless for 5 s after release, it was deemed to have entered tonic immobility. If it moved within 5 s, the procedure was repeated for a total of three times, and the number of induction attempts recorded. If it had not entered TI after three attempts, the bird was scored as “no TI induced”, and given a rightening time of 0. For the majority of birds entering TI, the observer used a stop watch to measure the time until first head movement (indicating that the bird had escaped the tonic immobility stage) and the time until rightening. A maximum time of 600 s was used, and if the bird had not rightened until then, the test was interrupted.

The TI recordings were made by two different observers, who calibrated their methods and techniques during a preceding pilot study on 20 unrelated birds. Since repeated TI:s are not valid (the birds habituate quickly), it was not possible to assess inter-observer reliability.

#### 2.3.3 Social dominance

At the age of 82–83 days, 58 birds were tested for their social dominance. The test was performed as described and validated previously [5]. Briefly, two weight-matched birds (not differing more than 10% in weight) of the same sex, one from each treatment, were placed in a test arena, measuring 1 x 1 m. The arena contained a feeder, which only allowed one bird at a time to feed. During five minutes, an observer recorded the time during which each of the two birds occupied the feeder. Within each pair, the bird monopolizing the feeder for the longest time was deemed to be the dominant, and the other the subdominant. All dominance observations were made by one and the same observer.

### 2.4 Sex hormone measurement

White leghorn chickens become sexually mature around 16 weeks of age, indicated by a marked increase in plasma 17-β-estradiol (E2, females) and testosterone (T, males) [27,28]. We therefore assessed the levels of these sex hormones in eight randomly selected birds of each sex and from each treatment (in total 32 birds) when they were 112 days old. Blood (0.5 ml/animal) was collected in a heparinized capillary tube following puncture of one wing vein, immediately centrifuged and then stored on ice until frozen for later analysis.

The hormones were analysed as described in detail earlier [2]. Briefly, after spiking with radioactive labelled hormone (T or E2) extraction was done with 100% EtOH. Following, incubation and centrifuging, the supernatant was dried and the pellet re-dissolved in PBSG. Average recoveries (± SD) were 81.4±3.2% for plasma T, and 76.1±4.9% for plasma E2. Steroid concentrations were then determined in one RIA with commercially available RIA kits: Coat-a-Count total testosterone (Siemens Medical Solutions Diagnostsics, Sweden) and Spectria Estradiol Sensitive RIA (Orion.Diagnostica AB, Sweden). The intra-assay coefficients of variation (CV) were 1.75% for plasma T, and 2.14% for plasma E2. The RIA results were corrected for recovery and initial sample volume. The cross-reactivity of the kits were below 1% for all related steroids.

### 2.5 Brain gene expression

Brain samples for gene expression analysis were taken on two occasions, from young (age 28 days) and adult (age 213 days) birds, from the groups assigned for brain sampling already at hatch and kept free from any behavioural tests. The young birds (24 ES and 24 C, balanced for sex) were sacrificed in conjunction with a physical restraint challenge as described previously [2]. Half of the chicks were culled immediately after being removed from the home pens for baseline measurement, while the other half was culled after being restrained in a tight cloth net for 30 min. The adult birds (16 ES and 16 C, balanced for sex) were culled immediately after being removed from the home pen. As mentioned above, these birds had not been exposed to any behavior tests during their lives, so the only difference between the ES and C birds used for brain gene expression analysis was the experience of the early stress. From the brains, the basal part of the midbrain, which consists mainly of the thalamus/hypothalamus region, was dissected using an established protocol in the lab [2] and snap-frozen in liquid nitrogen within 10 min of decapitation. All brains were dissected by one and the same experienced person, to ensure consistency across samples. The brain region was chosen for its central role in the regulation and control of the endocrine stress reaction. The 10 min time interval has previously been shown to be sufficiently short for preserving RNA for gene expression analysis [2].

Gene expression was analysed using Affymetrix GeneChip Chicken Genome Microarrays (with annotations based on Ensembl version 1, released May 2004), using standard procedures in GeneChip 3’ IVT Express Kit Users manual, as reported in detail previously [2]. Briefly, RNA was extracted with 1 ml TRIZOL (Ambion Inc.) per 50 mg sample from frozen and homogenized hypothalamus/thalamus tissue and mixed in sex-specific pools of three in the young and four in the adult subsamples, ensuring sufficient biological replicates from each treatment. RNA was precipitated with isopropanol, and quantity and quality was assessed with a NanoDop spectrophotometer and the Agilent 2100 Bioanalyzer. RNA integrity numbers were always over 8.0 for all samples.

Labeling and hybridization were performed at Uppsala Array Platform at Uppsala University, Sweden (http://www.medsci.uu.se/klinfarm/arrayplatform). The data was analysed in R/Bioconductor software environment (www.bioconductor.org). Normalization was done with the RMA method as detailed previously [2]. Microarray data has been uploaded to the ArrayExpress database (http://www.ebi.ac.uk/miamexpress/) with the accession numbers E-MTAB-924 and E-MTAB-3319.

### 2.6 Statistical analysis

Open Field data were sufficiently normally distributed (Anderson-Darling test P>0.05) to be analysed with general linear models (GLM), using treatment and sex as explanatory variables in the model. When there was a significant interaction, we tested the treatment effects separately within sex. Social dominance data were not normally distributed, and were analysed using non-parametric testing methods, as detailed in the results section. For tonic immobility, which contained censored data (where birds staid in tonic immobility for the maximum allowed test time), Kaplan-Meier survival analysis was used. This analysis does not allow full interaction models, so we tested the overall significance first, followed by analysis of each sex separately. For analysis of the social dominance, the significance of the proportion of dominant birds from the stressed birds was tested with a binomial test.

Hormonal data were log-transformed to reach sufficient normality before mean values were tested with an independent samples t-test.

For correlation of differential gene expression over age, we focused on the genes, which had the highest fold-change at each age. Fold-change was calculated as the log2-expression difference between stressed and control birds. We calculated this for young chicks both at baseline levels, as well as following the physical restraint. For the adults, only base-line values were obtained. We selected those genes which were found on the top-1000 list of highest fold-change at both ages, disregarding their p-values (the overlapping genes). Pearson correlation coefficients were then calculated for the fold-change values of those genes at the two ages. This analysis was made separately for each sex. The set of overlapping genes which showed a significant age-correlation were subjected to a Gene Ontology (GO) analysis, using the web-based software DAVID [29]. Since the focus of this experiment was to examine any lasting patterns in overall gene expression changes caused by early stress, we did not specifically assess biological variability within treatment groups, but relied on the correlation analysis outlined above.

All statistical tests were done with SPSS (v. 22).

## Results

### 3.1 Behaviour tests

**Fig 1:**
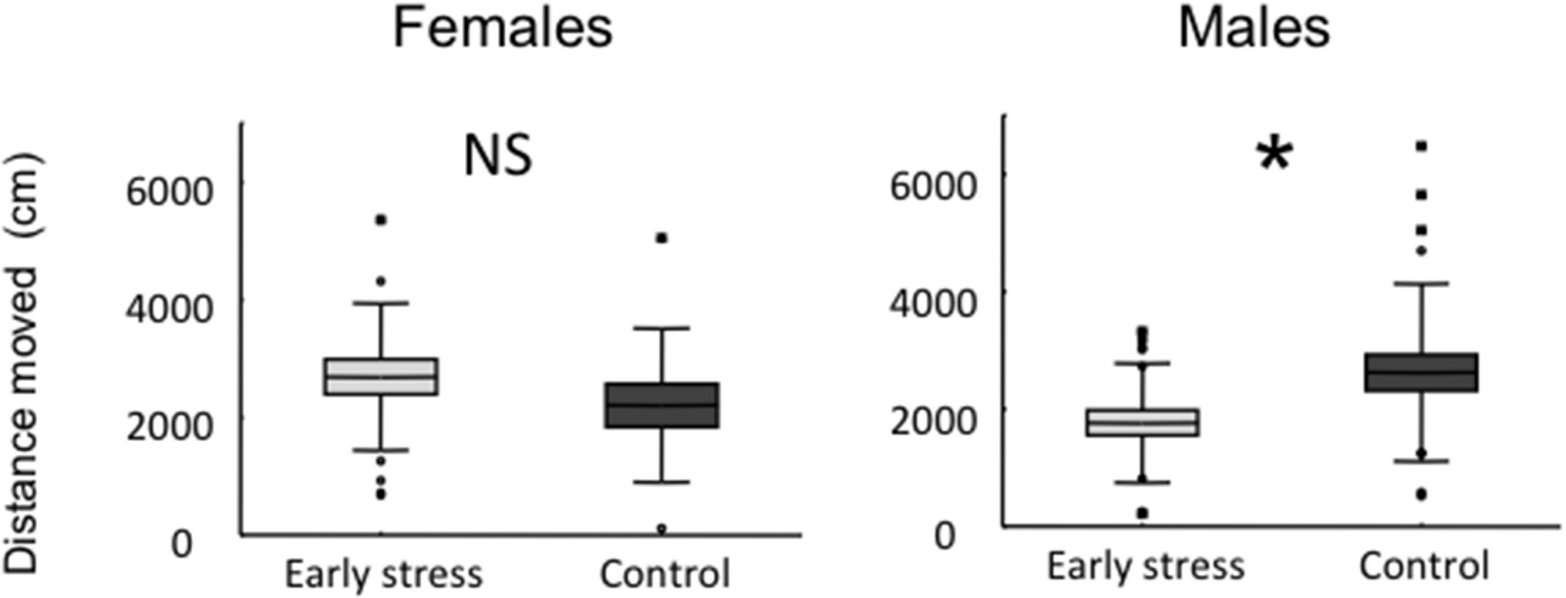
Box plots showing the Open Field activity for early stressed and control females (N = 29) and males (N = 44). The horizontal lines show average values, the boxes indicate standard errors, and the whiskers show standard deviation. Dots indicate outliers. A and B: Latency to leave the start zone (s); C and D: Total distance moved (cm). * : P< 0.05, NS = not significant.

There were no overall significant sex or treatment effects, and no interaction, on the latency to leave the start zone in the Open Field (Treatment: F_1, 69_ = 0.44, P = 0,51; Sex: F_1, 69_ = 0.02, P = 0.89; Treatment x Sex: F_1, 69_ = 1.19, P = 0.28). However, for total distance moved, there was a significant interaction between treatment and sex, although there were no overall effects (Fig 1; Treatment: F_1, 69_ = 0.35, P = 0,55; Sex: F_1, 69_ = 0.67, P = 0.41; Treatment x Sex: F_1, 69_ = 4.64, P = 0.03), where early stressed males, but not females, moved a shorter distance (females: F_1, 27_ = 1.0, P = 0.32; males: F_1, 42_ = 4.77, P = 0.03).

**Fig 2:**
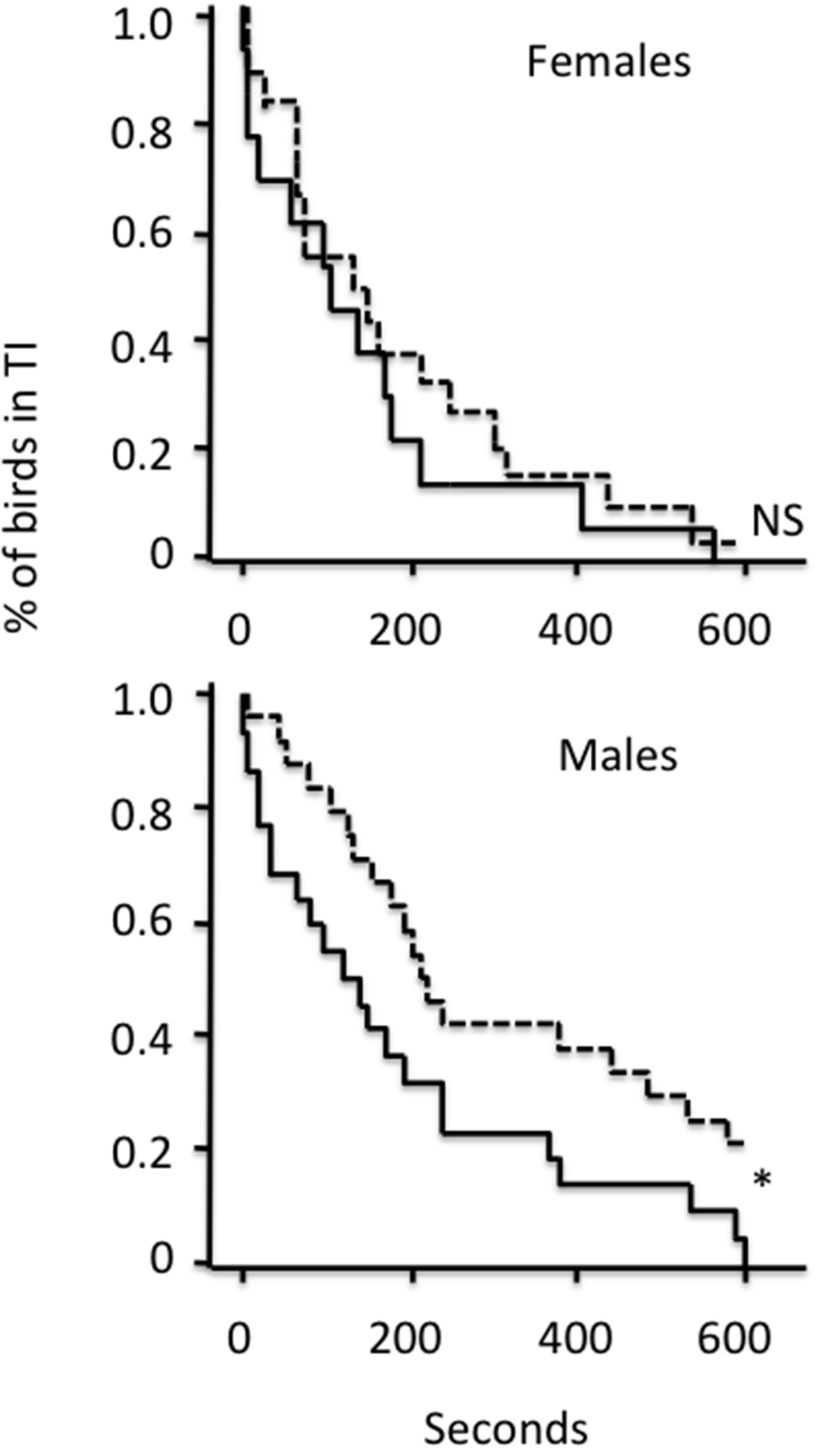
Survival plots for the variable “first head movement” in the tonic. immobility (TI) test. Females (N = 31) and males (N = 46) are shown in separate plots. Solid lines show values for early stressed birds, and dotted lines for control. The treatment effect is significant in males but not in females. * : P< 0.05, NS = not significant.

In the tonic immobility test, there were no overall effects of early stress or sex on the time to rightening, or on number of induction attempts (Kaplan-Meier survival analysis, X^2^(df = 1) = 1.74, P = 0.19), but there were significant effects on time to first head movement in males (Fig 2). Early stressed males moved sooner, hence stayed for a shorter time in tonic immobility (Kaplan-Meier survival analysis, X^2^(df = 1) = 5.39, P = 0.02). Again, in females there was no such effect (Kaplan-Meier survival analysis, X^2^(df = 1) = 0.45, P = 0.5).

**Fig 3:**
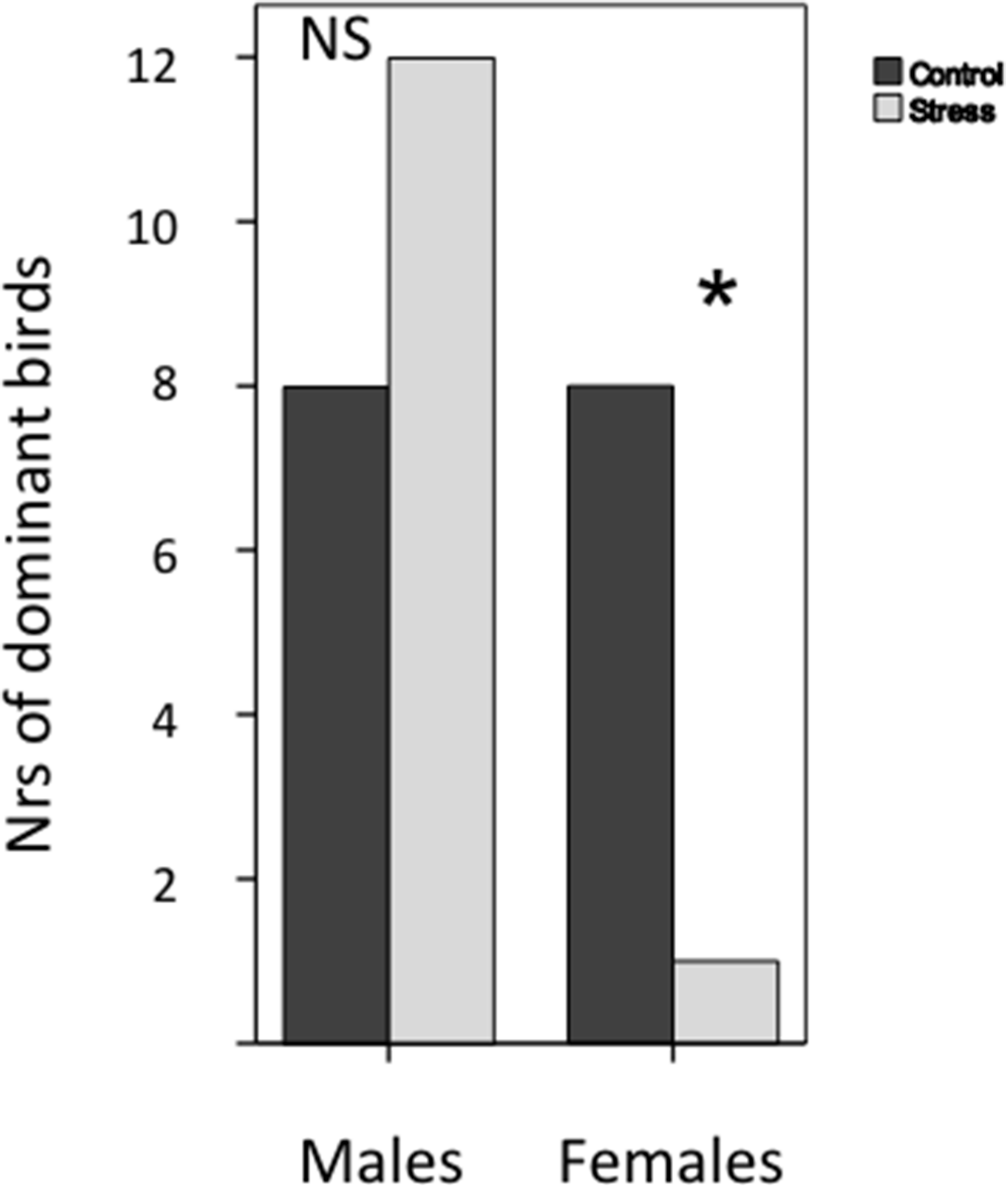
Social dominance in early stressed vs control male (N = 40) and female (N = 18) chickens. The figure shows the number of tested pairs in which either the early stressed or the control bird was scored as dominant. * : P< 0.05, NS = not significant.

In the social dominance test, significantly fewer early stressed females were dominant than control females (Fig 3; Binomial test, P = 0.039). In males, the proportion of early stressed males being dominant did not differ from that expected by chance (Binomial test, P = 0.50).

### 3.2 Hormones

**Fig 4:**
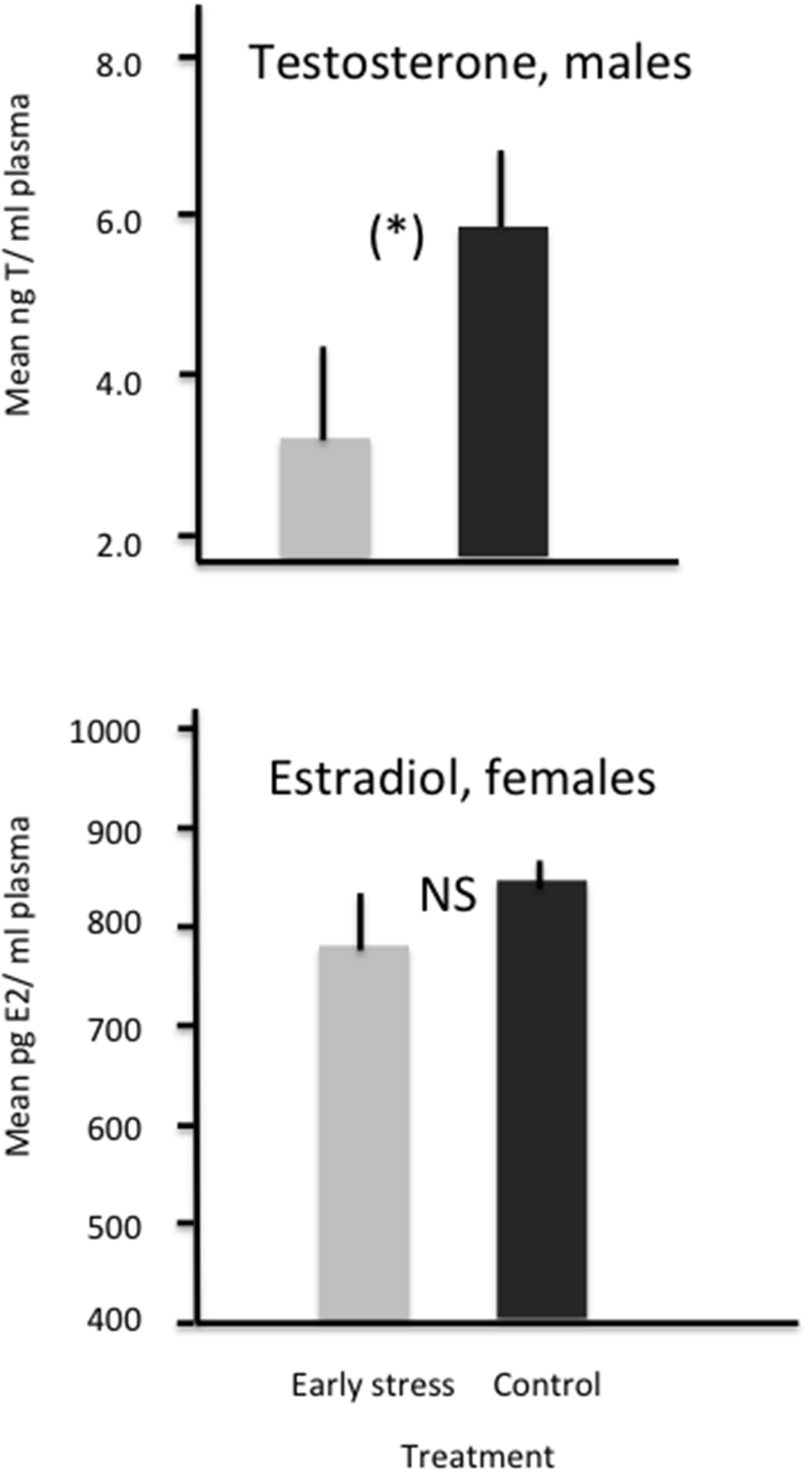
Mean (+/- SEM) plasma levels of testosterone in males (N = 16) and estradiol in females (N = 16) at 16 weeks of age. (*): P = 0.06, NS = not significant.

As seen in Fig 4, E2 in females at 16 weeks of age did not show any treatment effect (t_14_ = 1.25, P = 0.23). However, testosterone levels tended to be lower in early stressed males (t_14_ = 1.98, P = 0.06), indicating delayed sexual maturity in this group.

### 3.3 Gene expression

When comparing the baseline differential gene expression profiles (genes showing differential expression between treatment groups) of 28 days old chicks with that of 213 days old birds, there were 201 overlapping genes in the females (i.e., genes which were found on the top 1000 lists of fold change at both ages), and 202 in the males. In neither sex, there was any significant correlation of the fold change values between the ages.

**Fig 5:**
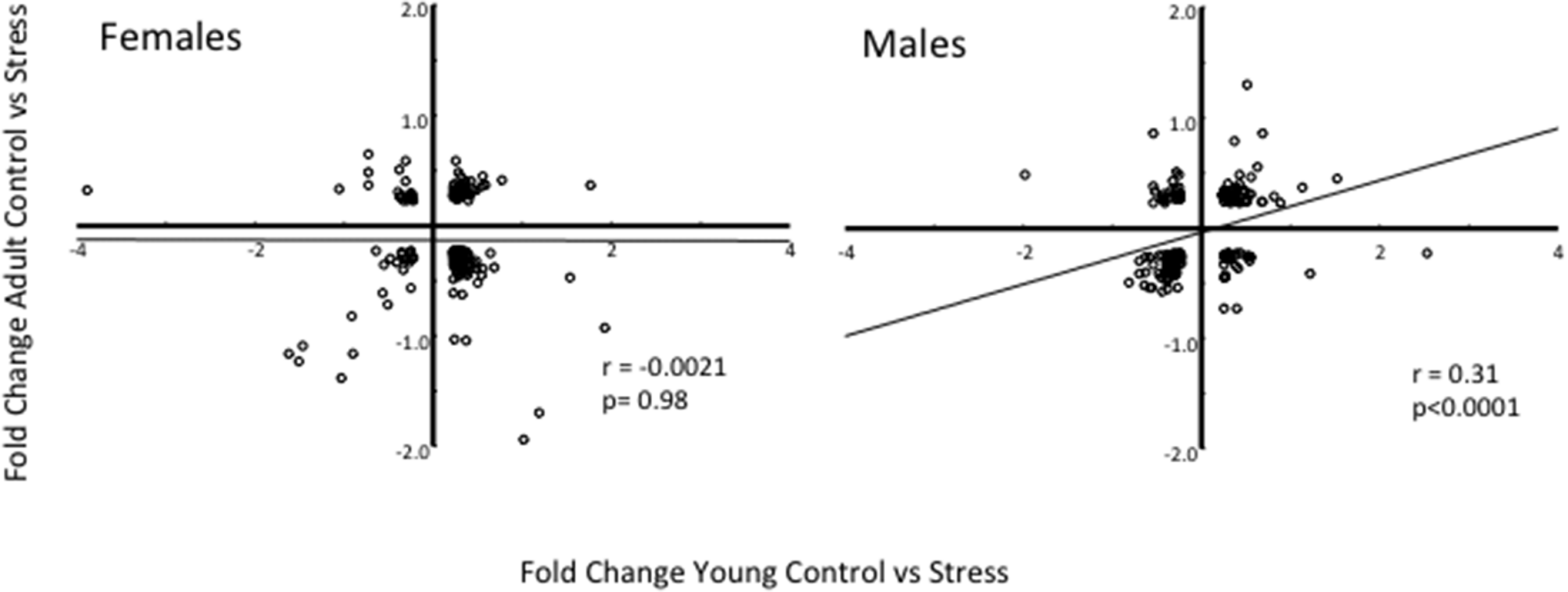
Correlation of differential expression of overlapping genes at 28 (young) and 213 (adult) days of age. The values of the axes represent the fold changes in the differential expression when comparing early stressed males and females. The plots show the correlations of the differential fold changes of the genes overlapping on the top 1000 differentially expressed at each age. The values for the young birds were obtained immediately after a 30 min physical restraint period as explained in Material and Methods, and in the adults they were obtained five minutes after capture and removal from the home pen.

Using the same adult differential gene expressions, but now compared to differential gene expression following physical restraint in the young birds, there were 166 and 199 overlapping genes respectively in the two sexes (Fig 5). In females, there was no correlation between the ages, but in males, the differential expression of the 199 overlapping genes at 28 and 213 days was significantly correlated. Hence in males, but not in females, the transcriptomic response to acute stress in young birds was significantly correlated to the gene expression profile of adult birds with a similar early stress experience. The full list of the overlapping genes in the males, with their annotations, fold changes and P-values can be found in S1 Table.

The gene ontology (GO) analysis, performed on the 199 overlapping genes showing a significant age correlation in males, produced 17 significantly enriched GO-terms (Table 1). Eight of these were specifically associated with neural development, such as neuron projection development, neuron development, axonogenesis, and neuron differentiation.

**Table 1:** Significantly enriched Gene Ontology (GO) terms among the 199 genes overlapping in the top 1000 list of differential gene expression in both 28 days old (post-restraint) and 213 days old male chickens.

Term	Count	PValue
GO:0031175~neuron projection development	6	<0.001
GO:0048666~neuron development	6	0.001
GO:0007409~axonogenesis	5	0.001
GO:0048812~neuron projection morphogenesis	5	0.002
GO:0030030~cell projection organization	6	0.002
GO:0048667~cell morphogenesis involved in neuron differentiation	5	0.002
GO:0048858~cell projection morphogenesis	5	0.003
GO:0032990~cell part morphogenesis	5	0.004
GO:0000904~cell morphogenesis involved in differentiation	5	0.005
GO:0007411~axon guidance	4	0.005
GO:0030182~neuron differentiation	6	0.007
GO:0006928~cell motion	6	0.008
GO:0000902~cell morphogenesis	5	0.013
GO:0032989~cellular component morphogenesis	5	0.021
GO:0007010~cytoskeleton organization	5	0.024
GO:0008045~motor axon guidance	2	0.045
GO:0000226~microtubule cytoskeleton organization	3	0.046

The table shows the GO-term assigned by the software DAVID, the number of genes in the category (Count) and the P-value (adjusted for false discovery rate) for the enrichment of the term.

## Discussion

The results showed that early stress caused sex-specific short- and long-term changes in behaviour, levels of gonadal hormonal levels and gene expression. The observed effects were mostly limited to males, except for social dominance, where females, but not males, were affected. Hence, a limited period of early daily stress exposure affects male and female chickens differently in a lifetime perspective.

Our results corroborate similar studies of sex differences in stress reactions in other species [10, 11]. The behavioural effects observed can be a result of several different possible mechanisms. For example, the HPA axis of male chickens appears to be more sensitive to central administration of corticotropin releasing hormone and arginine vasotocin, which causes a significantly higher corticosterone response to injections in males than in females [11]. Possibly, in the present study, the early stress treatment therefore caused a higher HPA-reaction in the male birds in the present study, with subsequent larger long-term effects. This may also be part of the reason why we found a possible effect on the levels of gonadal hormones in males, since the interaction between the HPA and the HPG axis is well known [36]. It should be noted that the hormonal analysis is based on few individuals, so the method may be underpowered to detect any true differences. Hence, care should be taken in interpreting these results. However, it should also be noted that the commercial laying strain used in our experiment is intensely selected for a uniform age at sexual maturity, so the results are most likely attributed to the early stress treatment.

The long-term behavioural and endocrine responses were accompanied by life-long correlated alterations in gene expression in males, but not in females. This can be a consequence of inherent variation between the sexes in this species, since it has been shown that sex differences in behaviour of the ancestral Red Junglefowl is closely related to stable epigenetic variation between males and females [6]. It is quite striking that eight out of the 17 significant GO terms linked to the genes showing consistent altered early stress responses were associated with neural processes.

Previous studies in a variety of species have shown that behavioral and endocrine effects of early stress often are related to lasting epigenetic changes in the brain [15,37], hence this could be the case also in our study. We did not attempt to unravel the exact effects on specific genes of the stress treatment, and therefore rely on the overall picture obtained through the Affymetrix Gene Expression microarray. To be able to be more specific regarding the effects on certain genes, the effects would have to be verified by an independent method, for example quantitative RT-PCR. However, the GO-terms associated with the overlapping genes are quite dissimilar from those involved in transgenerational effects of early stress, as shown by a comparison with earlier published data [2]. The reasons for this remain elusive, but it is possible that transgenerational effects are mediated by different genetic modifications than within-generation effects.

It should be noted that the gene expression analysis has clear limitations, and the results are therefore to be regarded mainly as suggestive. The fact that we pooled samples reduces the influence of individual outliers, but also limits the possibility to assess individual variation. Similarly to earlier research [2], we calculated correlation coefficients based on fold changes of overlapping genes, regardless of their p-values, which of course adds some uncertainty to the analysis. However, overlapping fold-change top lists should contain the major genes affected at both ages, and therefore, we believe that the correlations represent real effects.

The sex differences observed here are interesting, given the large differences in life history traits between male and female chickens. It would appear that an early stress experience programs the sexes for different life trajectories. In particular males seem to respond with developing a more protective and fearful behaviour phenotype, combined with a delayed sexual maturity. It is of course not possible to speculate on whether these reactions would be adaptive under natural conditions, but this is an interesting aspect, which should be followed up in future research.

However, it is also possible that the sex differences observed could be the result of, or could have been exacerbated by, modern breeding of egg laying chickens. There may have been a strong selection on resilience against early stress in female domesticated chickens, but less so in males, since only females are exposed to most of the extreme stress associated with commercial breeding, while the males are killed immediately after hatching. Female genotypes, which are able to cope with early stress, may therefore be more successful in commercial populations. This remains a speculative suggestion, but deserves some future research attention.

The methods used to induce stress and measure its consequences are mostly well established, but have not previously been combined into a single, comprehensive experiment like the present one. Integrating data on behaviour, physiology and gene expression provides a unprecedented broad picture of the long-term effects. We used an early stress treatment, resembling protocols used in rodents and other species where social isolation and maternal deprivation are considered severe stressors, and result in life-long phenotypic effects [30,31]. Although chickens are precocial birds, social isolation can be considered a stressor of a similar magnitude as maternal deprivation is in rodents, and studies of the closely related quail has shown that this type of protocol does indeed elicit a clear immediate stress response [32]. However, it is of course not possible to separate the effects of the different types of stressors, which were all imposed simultaneously on the chicks: handling, low temperature, social isolation, feed and water deprivation, and the intention of the present study was merely to create a situation of undisputable stress experiences early in the life of the birds.

For assessing levels of anxiety and fear following the early stress treatment, we used two different behaviour tests, commonly applied for this purpose, the Open Field test [26] and the Tonic Immobility test [26,33]. Red Junglefowl, ancestors of all domestic chickens, show a lower Open Field activity than domesticated egg layers [25], with significant sex differences [6], and also in the Tonic Immobility test, there are significant differences between Red Junglefowl and layers [34] and between male and female birds [6]. The present study adds important knowledge to previous results by showing that the response to each of these tests is modified by early stress experiences in males, but not in females. In addition, we assessed effects on later social dominance, using previously validated methods [5] [35]. In this test, early stressed females were significantly less dominant than control females, something not observed in males. There is a limitation in our studies in that we were not able to assess overall sex effects and sex * treatment interactions for some of the behavioral variables. Hence, further research would be needed to confirm our findings.

In conclusion, we found that in chickens, the two sexes show different long-term responses to stress encountered during the first weeks of life. Males showed higher levels of fear and anxiety, delayed sexual maturation and a consistently modified gene expression response to acute stress. Females, however, were more dominant in a food competition test.

## Supporting Information

S1 Table Overlapping genes in males. (XLSX)

S2 Table Dataset. (XLSX)
